# Impact of Mandibular Advancement Therapy on Occlusal Plane Orientation and Incisor Inclination in Obstructive Sleep Apnea Patients: A Retrospective Observational Study

**DOI:** 10.3390/jcm14238309

**Published:** 2025-11-22

**Authors:** Mauro Lorusso, Michele Tepedino, Francesca Papa, Graziano Montaruli, Fariba Esperouz, Rossella Luciano, Giuseppe Burlon, Mario Dioguardi, Lucio Lo Russo, Domenico Ciavarella

**Affiliations:** 1 Department of Clinical and Experimental Medicine, Dental School of Foggia, University of Foggia, 71112 Foggia, Italy; francesca_papa.571098@unifg.it (F.P.); graziano.montaruli@unifg.it (G.M.); fariba.esperouz@unifg.it (F.E.); rossella.luciano@unifg.it (R.L.); giuseppeburlon@gmail.com (G.B.); mario.dioguardi@unifg.it (M.D.); lucio.lorusso@unifg.it (L.L.R.); domenico.ciavarella@unifg.it (D.C.); 2 Department of Biotechnological and Applied Clinical Sciences, Dental School of L’Aquila, University of L’Aquila, 67100 L’Aquila, Italy; michele.tepedino@univaq.it

**Keywords:** obstructive, sleep apnea, mandibular advancement device, occlusal plane variations, upper incisor inclination

## Abstract

**Objective:** This observational study aimed to evaluate occlusal changes in patients with obstructive sleep apnea (OSA) treated with a mandibular advancement device after two years of therapy. **Methods:** Sixty adult patients with mild to moderate OSA (apnea–hypopnea index 15–30 events/h) were treated with the IMYS MAD for 24 months. Digital dental impressions were analyzed using Autodesk Meshmixer and Fusion 360 to measure sagittal, frontal, and occlusal angles, as well as upper incisor inclination at baseline (T0) and after treatment (T1). In addition, changes in the polysomnographic indices AHI and ODI were evaluated. Statistical analyses were performed using paired *t*-tests or Wilcoxon signed-rank tests depending on data normality (*p* < 0.05). **Results:** No significant differences were found between T0 and T1 in sagittal (IC 95% −2.053,1.433), occlusal (IC 95%, −1.202, 0.722) or frontal angles (IC 95% −1.487, 0.447), or in upper incisor inclination (IC 95%, 0.06,3.04). Polysomnographic parameters improved significantly, with mean AHI decreasing from 27.7 ± 12.3 (IC 95% 24.53–30.87) to 6.2 ± 4.0 events/h (IC 95% 5.17–7.27) and ODI from 19.7 ± 9.4 (IC 95% 17.27–22.13) to 4.7 ± 3.2 (IC 95% 3.82–5.48, *p* < 0.001). **Conclusions:** This study found that the IMYS MAD did not induce significant variations in occlusal plane orientation or upper incisors inclination after two years of treatment. These null findings suggest that the IMYS device may provide effective OSA management while minimizing the risk of occlusal or dental side effects. However, the retrospective design and the 24-month follow-up duration should be taken into consideration.

## 1. Introduction

Obstructive Sleep Apnoea Syndrome (OSAS) is a respiratory disorder characterised by recurrent episodes of partial or complete upper airway obstruction during sleep, associated with intermittent hypoxaemia and sleep fragmentation. The prevalence in the adult population ranges from 9% to 38% and tends to increase with age [1]. The predominant risk factors include obesity, male sex, craniofacial abnormalities, and the use of tobacco and alcohol [2,3,4]. In Europe, this syndrome has been increasingly recognised as a major public health issue, given its high prevalence and significant clinical and socioeconomic implications. Current epidemiological data estimate that approximately 44.3 million adults across the continent are affected, underscoring the substantial and growing burden this condition imposes on healthcare systems [5].

The pathogenesis of OSAS is complex and multifactorial, involving the interplay of anatomical, neuromuscular, and ventilatory factors [6,7]. Anatomical predispositions, such as craniofacial abnormalities, upper airway narrowing, or increased soft-tissue volume, reduce baseline pharyngeal patency. During sleep, the physiological decline in upper airway dilator muscle activity, particularly of the genioglossus further compromises airway stability and promotes pharyngeal collapse [8,9]. Ventilatory control instability, characterised by an elevated loop gain, may exacerbate these events by amplifying oscillations in breathing in response to small changes in carbon dioxide levels. In addition, abnormalities in the arousal threshold play a critical role, as individuals with a low threshold tend to wake prematurely in response to minimal respiratory effort, thereby limiting oxygen desaturation but perpetuating sleep fragmentation [10].

A variety of therapeutic strategies have been proposed for the management of this syndrome. Continuous positive airway pressure (CPAP) remains the gold standard for moderate to severe cases; nevertheless, its long-term clinical efficacy is often compromised by limited adherence [11]. The mandibular advancement device (MAD) has emerged as a valuable therapeutic alternative for patients with mild to moderate OSAS [12], as well as for those intolerant to CPAP, owing to its capacity to advance the mandible, enhance upper airway patency, and mitigate pharyngeal collapse [12].

Compared with previously investigated titratable mandibular advancement devices, the IMYS appliance presents unique biomechanical features. The device incorporates two vertical stainless-steel arms housed within superior slots that allow limited vertical freedom while preventing excessive mouth opening, potentially reducing constant tensile loading on dentoalveolar structures. In addition, the slot configuration enables controlled lateral mandibular motion, unlike many conventional MADs that restrict lateral excursions. This design aims to dissipate torsional and tipping forces that have been implicated in progressive changes in overjet, overbite, and occlusal-plane rotation during long-term MAD therapy.

Prolonged use of MADs results in repeated musculoskeletal and dentoalveolar stimulation due to the maintenance of the mandible in a protruded position during sleep. This condition generates orthodontic and orthopaedic forces that, over time, may lead to skeletal and dental modifications. Frequently reported alterations include proclination of mandibular incisors, retroclination of maxillary incisors, and a consequent reduction in overjet and overbite. These anterior changes are often accompanied by modifications in posterior occlusal contacts, which may contribute to alterations in the inclination of the occlusal plane. Furthermore, the chronic protrusive positioning of the mandible can induce neuromuscular adaptations of the elevator and protrusor muscles, influencing mandibular posture and occlusal dynamics [13].

Changes in the occlusal plane may exacerbate the clinical condition, as a clockwise rotation can significantly reduce the posterior airway space (PAS), thereby compromising airflow and potentially increasing disease severity [14]. Even minimal alterations in vertical dimension or mandibular rotation may shift the tongue and oropharyngeal structures posteriorly, contributing to airway collapse during sleep [15]. This highlights the importance of maintaining a stable occlusal environment to avoid unintended reductions in PAS, particularly in patients already predisposed to upper airway narrowing.

Furthermore, long-term use of mandibular advancement devices introduces progressive dental and skeletal adaptations that can influence occlusal stability [16]. Changes such as incisor proclination, molar intrusion or extrusion, and alterations in arch form may gradually modify occlusal relationships over time. These modifications can extend to the temporomandibular joints, potentially affecting disc position and joint loading [17,18]. As a result, some patients may become more susceptible to temporomandibular disorders, especially when pre-existing occlusal or skeletal imbalances are present. Continuous monitoring of occlusal changes is therefore essential to ensure the long-term functional and structural integrity of the stomatognathic system.

Nevertheless, the impact of MAD therapy on the occlusal plane remains relatively underexplored, despite its clear clinical relevance for both orthodontic and gnathological evaluation. A more detailed assessment of occlusal plane modifications in OSAS patients undergoing MAD treatment could enhance understanding of the long-term dentoskeletal consequences of this intervention, while also providing valuable insights for optimising appliance design and guiding comprehensive, multidisciplinary patient care. Accordingly, the present study aims to investigate alterations in the occlusal plane and maxillary incisor inclination in adult OSAS patients treated with MAD therapy. The null hypothesis is that long-term use of the IMYS device does not induce significant changes in occlusal plane orientation or upper-incisor inclination.

## 2. Materials and Methods

This retrospective study was conducted in accordance with the *Strengthening the Reporting of Observational Studies in Epidemiology* (STROBE) guidelines [19]. All procedures described in the study design complied with the ethical principles outlined in the Declaration of Helsinki and were approved by the Ethics Committee of the University. Retrospective data collection and analysis were performed with strict preservation of patient anonymity, and written informed consent was obtained from all participants. The inclusion criteria for patient selection were as follows: a diagnosis of OSA confirmed by polysomnographic examination; an apnea–hypopnea index (AHI) between 15 and 30 events per hour; an age range of 50 to 70 years; dental class I malocclusion; and ongoing treatment with MAD. Patients receiving continuous positive airway pressure (CPAP) therapy, those missing more than two teeth, and individuals with a history of otorhinolaryngologic or maxillofacial surgery were excluded from the study.

A priori power analysis was conducted using G*Power software (version 3.1.9.2; Franz Faul, University of Kiel, Kiel, Germany). Based on previous studies [20,21], the analysis indicated that a minimum of 45 participants was required to detect a large effect size (d = 0.5) using a paired *t*-test, with a significance level (α) of 0.05, and a statistical power (1 − β) of 0.95 [22].

Sixty patients (32 men and 28 women, mean age 62.5 years) treated at the Sleep Medicine Unit of the Dental Clinic, University of Foggia, between February 2020 and June 2022, were included in the study.

The patients included in the study were treated with the IMYS mandibular advancement device.

Each subject was evaluated at the beginning of treatment (T0) and after 24 months of MAD therapy (T1).

For each patient, changes in polysomnographic parameters, specifically, the apnea–hypopnea index (AHI) and the oxygen desaturation index (ODI) were assessed. Digital dental impressions were used to evaluate variations in the occlusal plane both in the sagittal and frontal dimensions, as well as changes in upper incisor inclination. The selected sample was analyzed using two different software programs: Autodesk Meshmixer (v. 3.5.474) and Autodesk Fusion 360 (v. 2603.1.15).

### 2.1. Autodesk Meshxmixer

**Figure 1:**
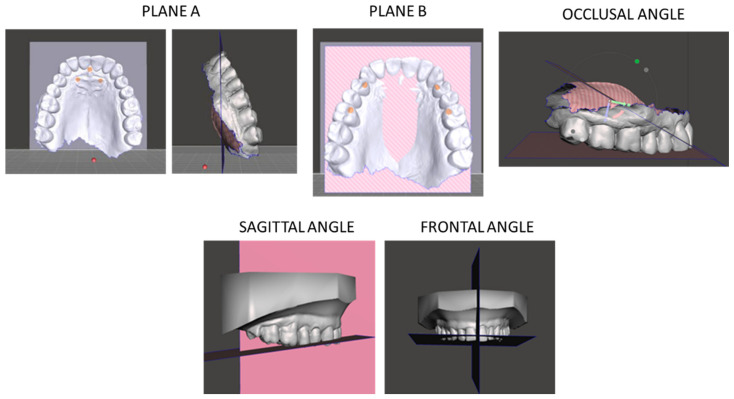
Planes A and B and occlusal, sagittal, and frontal angles.

The Autodesk Meshmixer platform is designed for the manipulation and analysis of 3D models. Using this software, it was possible to examine the maxillary arches of the patients included in the study. Specifically, the program allowed the creation of two reference planes intersecting predefined anatomical landmarks. The first plane (a) intersected two points located at the level of the palatal rugae and the incisive papilla, while the second plane (b) connected the points corresponding to the palatal cusps of the first maxillary premolars and the mesio-palatal cusps of the first maxillary molars. An additional model was then generated using the same maxillary arch, including plane *b* and a new median sagittal plane (c), which intersected the incisive papilla and extended along the mid-palatal suture. The intersection of these planes, forming another angle measured in the analysis, defined sagittal angle. Figure 1 illustrates planes A and B, as well as the corresponding occlusal, sagittal, and frontal angles.

**Figure 2:**
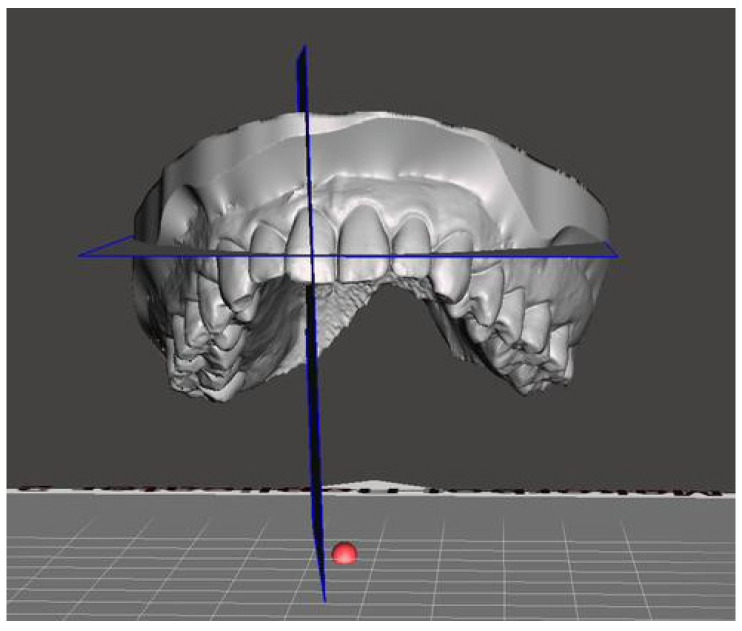
Inclination of the upper incisor.

The inclination of the upper incisors was calculated using the angle formed between the plane passing through the incisal papilla and the plane passing through the long axis of the incisor (Figure 2).

The models were then exported as STL files and analyzed using Autodesk Fusion 360 software to measure the obtained angles. All measurements were performed in a blinded manner and repeated twice by the same operator to minimize measurement error. Random error was calculated using Dahlberg’s formula (S = √∑d^2^/2N), where d represents the difference between the first and second measurements and *N* is the number of measurements evaluated [23,24]. The measurement error ranged between 0.01° and 0.03° for angular values. Intra-operator reliability was assessed using the intraclass correlation coefficient (ICC). The ICC values indicated good reliability for all measurements: occlusal angle (ICC = 0.912), sagittal angle (ICC = 0.915), frontal angle (ICC = 0.917), and upper-incisor inclination (ICC = 0.919).

Polysomnographic data were analyzed to assess the apnea–hypopnea index (AHI) and the oxygen desaturation index (ODI). Table 1 provides a schematic summary of the variables measured in the study.

### 2.2. IMYS Appliance

**Figure 3:**
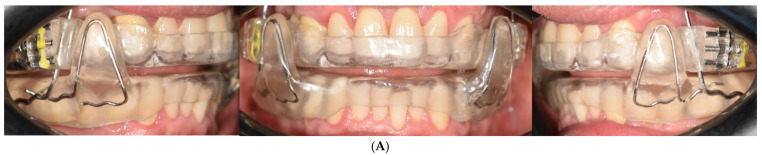

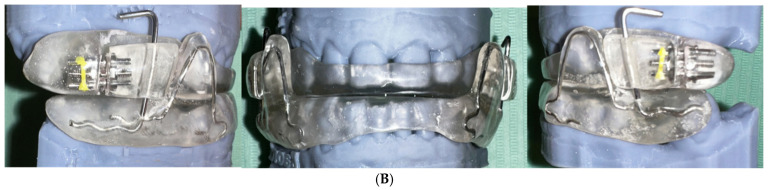
(**A**) Intraoral photographs showing frontal and lateral views of the IMYS; (**B**) Frontal and lateral views of the IMYS on resin models.

The device used is a customized and adjustable appliance called IMYS (It Makes You Sleep). It consists of two resin splints connected by two vertical stainless-steel bars and two lateral screws; the inclusion of these components enables the advancement of the device. The design of the vertical arms features a superior end inserted into a vertical slot mesial to the screw, within the resin of the upper splint, and an inferior end embedded in the resin of the lower splint (Figure 3). These arms allow limited vertical mandibular movement while preventing full mouth opening. Furthermore, the vertical slot housing the upper arm permits slight lateral movements of the mandible. To improve tongue positioning, a vertical palatal spot was added to the upper splint. Patients were instructed to wear the mandibular advancement device every night throughout the treatment period, ensuring a minimum continuous use of seven hours per night. Compliance was periodically verified through patient self-reports and monthly follow-up visit to confirm consistent appliance wear.

### 2.3. Statistical Analysis

Descriptive statistics were calculated, and the Shapiro–Wilk test was conducted to assess whether the data followed a normal distribution (Table 2). Effect sizes were calculated using standardized mean differences. To compare the measurements obtained before (T0) and after treatment (T1), either a paired *t*-test or a Wilcoxon signed-rank test was performed, depending on data distribution (Table 3 and Table 4). Statistical significance was set at *p* < 0.05.

## 3. Results

All participants completed the 24-month follow-up period, and no adverse events related to appliance wear were reported. No statistically significant differences were observed between baseline (T0) and post-treatment (T1) measurements for any of the evaluated parameters. The mean occlusal angle showed a minimal reduction from 28.80 ± 5.85° at T0 to 28.56 ± 5.98° at T1 (*p* = 0.694). Similarly, the sagittal angle decreased slightly from 44.73 ± 5.60° to 44.41 ± 3.22° (*p* = 0.452), while the frontal angle remained stable (90.11 ± 1.61° vs. 89.59 ± 3.34°, *p* = 0.694). The upper incisor inclination increased marginally from 33.4 ± 2.93° to 34.95 ± 3.86°, though this variation did not reach statistical significance (*p* = 0.175). Polysomnographic outcomes revealed robust and clinically meaningful improvements following mandibular advancement therapy. The AHI was reduced from 27.70 ± 12.30 to 6.22 ± 4.05 events/hour (*p* < 0.001), corresponding to a dramatic reduction in respiratory disturbance severity. Similarly, the ODI decreased from 19.70 ± 9.42 to 4.65 ± 3.21 (*p* < 0.001), reflecting a substantial enhancement in nocturnal oxygen saturation dynamics.

Effect size analysis revealed minimal pre–post changes across all angular measurements. The occlusal angle showed a negligible effect (d = −0.06), consistent with the narrow 95% CI (−1.20 to 0.72). Similarly, the sagittal angle presented an effect size of −0.04, with a CI ranging from −2.05 to 1.43. The frontal angle demonstrated a small negative effect (d = −0.14), with a CI of −1.49 to 0.45. Only the upper incisor inclination showed a slightly larger, yet still small, effect size (d = 0.26), corresponding to a CI of 0.06 to 3.04. Overall, all effect sizes fell within the small-to-negligible range, indicating minimal angular variation between pre- and post-treatment assessments.

No correction for multiple comparisons was applied, as none of the tested variables reached statistical significance. Under these conditions, applying a multiple-comparison adjustment would not have altered the interpretation of the results and was therefore deemed unnecessary, given the absence of any inflation of the Type I error rate.

## 4. Discussion

The mandibular advancement device is a removable intraoral appliance designed for therapeutic use in the management of obstructive sleep apnea and primary snoring. It functions mechanically by repositioning the mandible in a protrusive position relative to its habitual occlusal relationship defined centric relation or resting position, thereby improving the patency of the upper airway during sleep [25]. The pathophysiological principle underlying the use of MAD is based on mandibular advancement, which in turn produces a forward displacement of the tongue base and oropharyngeal soft tissues, thereby reducing upper airway collapse during sleep. This mechanism improves airway patency and decreases both the frequency and severity of apnea and hypopnea events [26]. Moreover, the mandibular advancement effect may contribute to improving the neuromuscular tone of the pharyngeal muscles and to stabilizing the anatomical structures involved in nocturnal airway collapse. It has also been reported that MAD therapy may contribute to improved cardiovascular function, as reflected by reductions in heart rate and a decreased risk of severe chronic complications, particularly in individuals with cardiovascular disease [27]. As highlighted in previous investigations focused on clear aligners, alterations in the occlusal plane and the curve of Spee may arise during aligner therapy, even when no vertical tooth movements, such as extrusion or intrusion, are intentionally planned [28,29]. Furthermore, several factors, such as arch form and growth pattern, as well as occlusal force and molar relationship, may influence occlusal stability and, consequently, the overall effects of treatment [30,31].

The present study aimed to evaluate potential dentoskeletal and occlusal changes in OSA patients treated with a MAD, focusing on variations in sagittal, occlusal, and frontal angles, as well as upper incisor inclination. After 24 months of therapy, no statistically significant differences were observed in any of these parameters. The results indicate that treatment with the IMYS does not induce modifications in the occlusal plane or in upper incisor inclination, suggesting a high degree of biomechanical stability over the two-year period. From a biomechanical perspective, the absence of angular and dental changes observed in the present study may also be explained by the mechanical features of the IMYS device. Its dual-splint configuration, connected through vertical stainless-steel bars and lateral screws, allows for controlled vertical and lateral mandibular movement. Such mobility can minimize the transmission of constant torsional stress to the dentoalveolar structures, thereby limiting occlusal plane rotation or incisor torque alterations. This contrasts with earlier monobloc designs, or those requiring advancement elastics, which exerted rigid and uneven forces and were more likely to produce progressive occlusal changes over time. This finding is consistent with previous studies reporting that well-designed, titratable MAD cause minimal or no occlusal alterations when properly adjusted and monitored. In a four-year follow-up study, Ishida et al. [21] reported a decrease in upper incisor inclination and overjet in patients treated with MAD.

Doff et al. [32] and Marklund et al. [33] reported similar dentoalveolar effects during MAD therapy, with reductions in overjet and overbite related to retroclination of the upper incisors and proclination of the lower incisors, while skeletal parameters remained stable. A meta-analysis by Chen et al. [34] confirmed that these changes tend to develop progressively over long-term use, usually beyond three years and are typically considered clinically acceptable when appliance titration is properly controlled. Although a mild clockwise mandibular rotation has been described, major skeletal alterations were not observed. In comparison, Linsen et al. [35] noted three-dimensional tooth movements, including mesial occlusion, anterior open bite, and reduced occlusal contacts; however, these reflected mainly tipping movements rather than true bodily displacement and were judged to have limited clinical relevance. Overall, our null angular findings are consistent with evidence indicating that measurable dentoalveolar adaptations typically arise only after long-term MAD use.

Table 5 provides a comparative overview of key characteristics from previous investigations, including follow-up duration, device type, and the principal dento-occlusal changes reported.

Maintaining the stability of the occlusal plane is particularly important in adult patients, since even minor alterations may induce adaptive or pathological changes in the temporomandibular joint.

Camacho-Alvarez et al. [36] reported an association between occlusal plane inclination and certain condylar movements, whereas Raja et al. [37] did not identify significant relationships between occlusal inclination and joint-related parameters. Given these inconsistent findings, any potential link between occlusal plane orientation and temporomandibular joint behavior should be regarded as a theoretical consideration rather than a conclusion supported by direct evidence. However, caution is warranted, as the protrusive action of mandibular advancement devices may induce muscular adaptations that could influence TMJ function, particularly when occurring in combination with changes in occlusal plane orientation.

The IMYS architecture, with controlled vertical and lateral freedom, likely dissipates torsional and tipping forces, reducing moments that would otherwise promote occlusal plane rotation or incisor torque changes. Moreover, the titration strategy adopted in this cohort appears conservative (as indirectly suggested by the robust AHI/ODI improvement without the need for aggressive adjustments), which may have limited cumulative dental loading. Notably, the measurement protocol showed excellent reliability (ICC > 0.90; very low Dahlberg error), supporting that the null findings reflect true stability rather than imprecision. Finally, although the follow-up period may theoretically be insufficient to detect potential changes in occlusal plane inclination, this appears unlikely, as such modifications are generally considered adaptive responses of the occlusal and neuromuscular system and typically manifest within a few months of appliance use.

Clinically, our results indicate that the IMYS device may provide effective OSA management while maintaining occlusal plane inclination and upper incisor position over a two-year period, suggesting a limited risk of dento-occlusal side effects. These findings may offer reassurance to practitioners when considering IMYS for long-term therapy. The IMYS device appears biomechanically stable for 2 years, but that minor, cumulative dental changes beyond this timeframe remain plausible. Although no signs suggestive of temporomandibular dysfunction were observed in the present cohort, even subtle occlusal plane or incisor inclination changes may theoretically influence TMJ loading patterns over time. Nonetheless, larger prospective cohorts, extended follow-up durations, and comparative evaluations with other MAD designs are warranted to confirm these observations and further elucidate the long-term biomechanical behavior of this appliance.

### Limitations of the Study

Although the 24-month follow-up provides clinically meaningful information, the relatively limited sample size may not fully reflect long-term dentoskeletal adaptations that could emerge after prolonged use. Moreover, the lack of a control group, either untreated individuals or patients treated with alternative MAD, limits external comparison, and the present findings should therefore be interpreted as within-subject changes only. The absence of sex-stratified analyses might mask differential adaptations between males and females. In addition, angular measurements were obtained in two dimensions from digital models and may not detect subtle three-dimensional variations that would be observable with CBCT.

Future studies including larger cohorts, extended follow-up, appropriate control groups, sex-stratified analyses, and full 3D imaging will be essential to confirm and expand these findings.

## 5. Conclusions

The IMYS MAD produced significant polysomnographic improvements without inducing measurable dentoalveolar or occlusal changes over 24 months, supporting its biomechanical safety in medium-term OSA management. These findings suggest that the IMYS device is both effective and biomechanically safe for the medium-term management of OSA, providing improvements in polysomnographic indices while minimizing the risk of undesirable occlusal and dental side effects frequently associated with prolonged MAD therapy.

This stability has relevant clinical implications, indicating that IMYS may be considered a stable and patient-friendly MAD option in medium-term management of OSA.

## Figures and Tables

**Table 1:** Summary of measured variables.

Variable	Definition	Reference Planes
Occlusal angle	Angle formed by the intersection of two reference planes: a–b	Plane a: incisive papilla and palatal rugae; Plane b: palatal cusps of first maxillary premolars and mesio-palatal cusps of first maxillary molars
Sagittal angle	Angle formed by plane b and c	Plane b, Plane c: mid-palatal sagittal plane through incisive papilla
Frontal angle	Angle measured between by plane b and c on frontal plane	Plane b and c in frontal projection
Upper incisor inclination	Angle between long axis of upper central incisor and reference plane	Incisive papilla plane and long incisor axis

**Table 2:** Descriptive statistics and normality test results for variables taken at T0 and T1.

Variables	Time	Mean (°)	SD (°)	Median (°)	Min (°)	Max (°)	Passed Normality Test
Occlusal angle	T0	28.8	5.85	30.2	19.2	35.7	No
Occlusal angle	T1	28.56	5.98	29.1	17.1	37.3	No
Occlusal angle	Δ (T1 − T0)	−0.24	3.8	−0.25	−6.3	−6.0	Yes
Sagittal angle	T0	44.73	5.6	46.1	30.4	51.7	No
Sagittal angle	T1	44.41	3.22	43.9	39.0	49.5	No
Sagittal angle	Δ (T1 − T0)	−0.31	6.89	−1.65	−10.4	17.3	No
Frontal angle	T0	90.11	1.61	89.8	87.2	92.5	No
Frontal angle	T1	89.59	3.34	90.1	81.0	93.5	No
Frontal angle	Δ (T1 − T0)	−0.52	3.82	−0.1	−8.0	4.8	No
Upper incisors inclination	T0	33.4	2.93	33.3	28.6	39.6	Yes
Upper incisors inclination	T1	34.95	3.86	34.75	29.2	44.5	No
Upper incisors inclination	Δ (T1 − T0)	1.55	5.89	2.1	−10.4	12.9	Yes

**Table 3:** Descriptive statistics and *t*-test or Wilcoxon test of AHI and ODI before (T0) and after (T1) treatment.

Variables		Mean	Standard Deviation	95%CI	*p*
AHI	T0	27.70	12.30	24.53–30.87	0.001
	T1	6.22	4.05	5.17–7.27	
ODI	T0	19.70	9.42	17.27–22.13	0.001
	T1	4.65	3.21	3.82–5.48	

**Table 4:** Wilcoxon signed-rank test or paired *t*-test used to compare pre- and post-treatment values for occlusal, sagittal, and frontal angles, as well as upper incisor inclination.

Angle	Mean Pre-Treatment	Mean Post-Treatment	*p*	Statistic	df	95% CI	Effect Size
Occlusal *	28.80 ± 5.85°	28.56 ± 5.98°	0.694	t = 0.328	59	−1.2, 0.72	−0.06
Sagittal	44.73 ± 5.6°	44.41 ± 3.22°	0.452	Z = −0.53	-	−2.05, 1.43	−0.04
Frontal	90.11 ± 1.61°	89.59 ± 3.34°	0.820	Z = −0.16	-	−1.49, 0.45	−0.14
Upper * incisors inclination	33.4 ± 2.93°	34.95 ± 3.86°	0.175	t = −1.392	59	0.06, 3.04	0.26

* *t*-test.

**Table 5:** Comparative Summary of Previous Studies on MAD-Induced Changes.

Study	Duration	Device Type/Description	Reported Changes
Ishida et al. [21]	4 years	Custom titratable mandibular advancement device	Reduction in upper incisor inclination and overjet
Doff et al. [32]	2 years	Adjustable two-piece MAD	Reduction in overjet and overbite; lower incisor proclination; upper incisor retroclination; no skeletal changes
Marklund et al. [33]	2.5 ± 0.5 years	Protrusive oral appliance	Reduction in overjet and overbite; minor dentoalveolar effects
Chen et al. [34]	>3 years (meta-analysis)	Various long-term MADs	Reduction in overjet and overbite; upper incisor retroclination; lower incisor proclination; stable skeletal pattern; clockwise mandibular rotation
Linsen et al. [35]	T1: 11.9 ± 7.1 months T2: 31.9 ± 25.4 months	Wing-type vs. thrust-type MADs	3D tooth movement; mesial occlusion; anterior open bite; decreased occlusal contacts

## Data Availability

The data presented in this study are available on request from the corresponding author due to privacy restriction.
